# NMR ^1^H, ^13^C, ^15^N backbone resonance assignments of the T35S and oncogenic T35S/Q61L mutants of human KRAS4b in the active, GppNHp-bound conformation

**DOI:** 10.1007/s12104-021-10050-7

**Published:** 2021-10-22

**Authors:** Alok K. Sharma, Marcin Dyba, Marco Tonelli, Brian Smith, William K. Gillette, Dominic Esposito, Dwight V. Nissley, Frank McCormick, Anna E. Maciag

**Affiliations:** 1 NCI RAS Initiative, Cancer Research Technology Program, Frederick National Laboratory for Cancer Research, Leidos Biomedical Research, Inc., Frederick, MD 21701 USA; 2 National Magnetic Resonance Facility at Madison, Biochemistry Department, University of Wisconsin–Madison, Madison, WI 53706 USA; 3 Helen Diller Family Comprehensive Cancer Center, University of California, San Francisco, CA 94158 USA; 4 Leidos Biomedical Research, Inc., Post Office Box B, Frederick, MD 21702 USA

**Keywords:** GTPase KRAS, GppNHp, HSQC, T35S, Q61L, CSP

## Abstract

RAS proteins cycling between the active-form (GTP-bound) and inactive-form (GDP-bound) play a key role in cell signaling pathways that control cell survival, proliferation, and differentiation. Mutations at codon 12, 13, and 61 in RAS are known to attenuate its GTPase activity favoring the RAS active state and constitutively active downstream signaling. This hyperactivation accounts for various malignancies including pancreatic, lung, and colorectal cancers. Active KRAS is found to exist in equilibrium between two rapidly interconverting conformational states (State1–State2) in solution. Due to this dynamic feature of the protein, the ^1^H–^15^N correlation cross-peak signals of several amino acid (AA) residues of KRAS belonging to the flexible loop regions are absent from its 2D ^1^H–^15^N HSQC spectrum within and near physiological solution pH. A threonine to serine mutation at position 35 (T35S) shifts the interconverting equilibrium to State1 conformation and enables the emergence of such residues in the 2D ^1^H–^15^N HSQC spectrum due to gained conformational rigidity. We report here the ^1^H^N^, ^15^N, and ^13^C backbone resonance assignments for the 19.2 kDa (AA 1–169) protein constructs of KRAS-GppNHp harboring T35S mutation (KRAS^T35S/C118S^-GppNHp) and of its oncogenic counterpart harboring the Q61L mutation (KRAS^T35S/Q61L/C118S^-GppNHp) using heteronuclear, multidimensional NMR spectroscopy at 298 K. High resolution NMR data allowed the unambiguous assignments of ^1^H–^15^N correlation cross-peaks for all the residues except for Met1. Furthermore, 2D ^1^H–^15^N HSQC overlay of two proteins assisted in determination of Q61L mutation-induced chemical shift perturbations for select residues in the regions of P-loop, Switch-II, and helix α3.

**Supplementary Information:**

The online version contains supplementary material available at 10.1007/s12104-021-10050-7.

## Biological context

*RAS* is the most frequently mutated oncogene in cancer. RAS proteins exhibit a molecular “on” and “off” cycling mechanism to render RAS in its active form (GTP-bound), and in its inactive form (GDP-bound), respectively. Of the three major isoforms of RAS, mutant KRAS protein plays a causal role in three major human cancers: lung, colorectal, and pancreas (Bos 1989). Intrinsic GTP hydrolysis rates of RAS proteins are slow and require interaction with GTPase activating proteins (GAPs) to stimulate GTP hydrolysis (Bos et al. 2007). Mutations at codons 12, 13, and 61 disrupt GAP-mediated GTP hydrolysis, causing these mutants to accumulate in the active, GTP-bound conformation and promoting cell proliferation. Codon 61 (Q61) mutants exhibit the slowest intrinsic GTP hydrolysis rates among oncogenic KRAS mutants (Hunter et al. 2015). This mutation also accelerates the rate of GDP-to-GTP exchange, leading to accumulation of the Q61 mutant protein in the active state.

Structurally, KRAS is composed of an N-terminal cytoplasmic guanosine nucleotide-binding domain (G domain of ~ 1–169 AA), and a C-terminal hypervariable region (comprising ~ 19 AA) anchored to the cell membrane. The canonical RAS members (HRAS, KRAS, and NRAS) share highly conserved structural elements within the G domain. These structural elements include the flexible regions of P-loop (AA 10–17) that binds nucleotide phosphates, and Switch-I (SW-I; AA 25–40) and Switch-II (SW-II; AA 57–75) that interact directly with effector proteins, such as Raf, PI3K, and RalGDS, and the guanine nucleotide exchange factors (GEFs). However, the majority of residues from the flexible P-loop, SW-I, and SW-II regions are undetectable in the 2D ^1^H–^15^N HSQC spectrum of GTP-bound KRAS within/near physiological pH range (Hu and Redfield 1997; Ito et al. 1997; Buhrman et al. 2011; Araki et al. 2011). The absence of these residues from the spectrum is closely linked to the intrinsic dynamic features of regional polysterism that allows the RAS protein to exist in equilibrium between at least two highly interconverting states: State1 and State2 conformations, also recognized as open (effector binding deficient) and closed (effector binding enabled) conformations, respectively (Ito et al. 1997; Araki et al. 2011). These missing structural regions play an important role in constituting the protein–protein interaction interface of RAS with effector proteins, and, potentially, in forming the binding site(s) for novel small molecule inhibitors.

Residue Thr35 is closely coupled to the Switch function (Spoerner et al. 2001). The T35S (Thr35Ser) mutation in KRAS-GTP is known to shift the native State1-State2 equilibrium to the State1 conformation, resulting in the rigidification of the structural conformations of P-loop, SW-I and SW-II. Such conformational change enables the emergence of ^1^H–^15^N correlation cross-peaks from these flexible regions in the 2D ^1^H–^15^N HSQC spectrum. T35S KRAS-GTP, therefore, could be used for the detection of ligand-binding induced residue perturbations for all the backbone amide signals, and thus, unambiguous determination of ligand binding site(s). Availability of NMR analysis becomes important in the discovery pipeline, especially for those inhibitors that upon binding shift the equilibrium conformation of KRAS-GTP to the “State1 like” conformation. In a G12D KRAS-GTP ligand screening campaign reported recently, the T35S mutation was found important in reducing the likelihood of the protein being constitutively active in the protein-complexes (Zhang et al. 2020).

We applied NMR methods to understand the structural differences between the wildtype (WT) and a Q61 oncogenic mutant of KRAS-GppNHp in solution. We present here, NMR backbone assignments and secondary structures of the T35S/C118S mutant of KRAS4b(1–169)-GppNHp (named KRAS^T35S/C118S^-GppNHp hereafter), and of the Q61L oncogenic mutant T35S/Q61L/C118S KRAS4b(1–169) (named KRas^T35S/Q61L/C118S^-GppNHp hereafter). From these assignments we determine the residues that elicit the Q61L-induced conformational changes in the protein.

## Methods and experiments

### Protein expression and purification

#### Cloning

Gateway Entry clones for KRAS4b(1–169) T35S/C118S and KRAS4b(1–169) T35S/Q61L/C118S were generated by standard cloning methods and incorporate an upstream tobacco etch virus (TEV) protease cleavage site (ENLYFQG) followed by the appropriate KRAS sequences. Sequence validated Entry clones were sub-cloned into pDest-566, a Gateway Destination vector containing a His6 and maltose-binding protein (MBP) tag to produce the final *E. coli* expression clones (Taylor et al. 2017). The BL21 STAR (*rne131*) *E. coli* strain containing the DE3 lysogen and rare tRNAs (pRare plasmid, Cm^R^) was transformed with the expression plasmids (Amp^R^).

#### ^13^C/^15^N or ^15^N isotopic labeling of KRAS4b(1–169)

Seed cultures were inoculated from glycerol stocks of the transformed strains into 300 mL of the basal medium and buffer of Studier’s MDAG135 medium in a 2 L baffled shakeflask for 16 h at 37 °C until mid-log growth. In the interim, 15 L of ModM9 medium (2 g/L ^13^C glucose as needed, 1 g/L ^15^N-NH_4_Cl, 2 mM MgSO_4_, 100 μM CaCl_2_, 4 μM ZnSO_4_, 1 μM MnSO_4_, 4.7 μM H_3_BO_3_ and 0.7 μM CuSO_4_) was prepared in a 20 L Bioflow IV bioreactor (Eppendorf/NBS). ^15^N-labeled protein was expressed using ^15^N-NH_4_Cl and ^12^C-glucose as sole N and C sources in the expression medium. When the seed culture reached mid-log phase, the culture was removed, centrifuged at 3000×*g* for 10 min at 25 °C, the pellet resuspended with 100 mL of ModM9 medium pulled from the bioreactor, and returned to the bioreactor as inoculum. The culture was grown at 37 °C with agitation of 350 RPM. When the OD600 reached 0.4–0.6 (~ 3 h), IPTG was added to a final concentration of 500 μM and the temperature adjusted to 16 °C. After 21 additional hours of growth the cells were collected by centrifugation (5000×*g* for 10 min at 4 °C). Cell pellets were immediately frozen at − 80 °C.

#### Purification

KRAS4b(1–169) proteins were purified as outlined (Kopra et al. 2020). Briefly, the expressed proteins of the form His6-MBP-tev-POI, were purified from clarified lysates by IMAC, treated with His6-TEV protease to release the target protein of the form Gly-KRAS4b(1–169). The target protein was separated from other components of the TEV protease reaction by a second round of IMAC. Final protein was subsequently buffered exchanged by preparative SEC. Protein constructs were GppNHp loaded by using an exchange reaction in KRAS buffer (20 mM HEPES, pH 7.3, 150 mM NaCl, 2 mM MgCl_2_, and 1 mM TCEP). Briefly, 30 molar excess of GppNHp solution to protein (GppNHp tetralithium salt, Jena Biosciences, Jena, Germany; NU-401-50) and 2 M (NH_4_)_2_SO_4_ solution were prepared in KRAS buffer. (NH_4_)_2_SO_4_ was added to protein sample to achieve 200 mM final concentration. Subsequently a total of 10% of GppNHp stock solution was mixed with protein sample. Alkaline phosphatase on agarose beads (Sigma-Aldrich, MO, USA) were added to the reaction mixture (1 unit of alkaline phosphatase per mg of protein). Reaction was allowed to occur at RT for 1.5 h. Subsequently, the alkaline phosphatase on agarose beads was removed by filtration using 0.22 µm Millex-GP 33 mm syringe filters (Millipore Sigma, MA, USA). Remaining 90% of stock GppNHp solution was then mixed with agarose free sample of KRAS protein and set for incubation for next 30 min. 0.22 Micron filtered sample was then FPLC purified from low molecular components using 5 × 5 mL (each 16 × 25 mm) Cytiva HiTrap desalting columns (Global Life Sciences Solutions USA LLC, MA, USA). A total of 3 mL sample (a maximum of 15 mg protein per injection) was injected into 5 mL loop for each run. All purified proteins showed apparent purity of > 95% as detected by Coomassie Blue Staining after SDS-PAGE. The GppNHp-bound state of the purified protein was ascertained by HPLC and mass spectrometry. Sample concentration was measured using a NanoDrop One microvolume UV–Vis spectrophotometer (Thermo Fisher Scientific, MA, USA).

#### NMR spectroscopy

^13^C/^15^N-labeled and ^15^N-labeled samples of 0.8 mM concentration were prepared for KRAS^T35S/C118S^-GppNHp and KRAS^T35S/Q61L/C118S^-GppNHp in a solvent composition of 93% H_2_O/7% D_2_O that contained 20 mM MES-d_13_ (pH 6.5; DLM 4363, CIL), 50 mM NaCl, 100 mM KCl, 1 mM TCEP-d_16_, 2 mM MgCl_2_, 100 µM 2,2-dimethyl-2-silapentanesulfonic acid (DSS) as internal standard, and 0.02% (w/v) NaN_3_ to avoid any unwanted bacterial growth over time. A total of 320 μL sample volume was used for each sample in a standard Shigemi tube.

NMR experiments were carried out on Bruker Avance 700 MHz spectrometer equipped with a 5-mm TCI cryoprobe (*in-house*) and on a Bruker Avance III HD 750 MHz spectrometer equipped with a 5-mm TXI (Z-axis gradient) cryoprobe (NMRFAM). All data were collected at 298 K in the gradient-selected sensitivity-enhanced mode. To accomplish backbone ^1^H^N^, ^15^N, ^13^C^α^, ^13^C^β^, and ^13^CO assignments, NMR data of 1D ^1^H, 2D ^1^H–^15^N HSQC, and triple-resonance experiments of HNCACB, CBCA(CO)NH, HNCA, HN(CO)CA, and HNCO were collected. For KRAS^T35S/Q61L/C118S^-GppNHp the HN(CA)CO was also collected. Assignments were further validated in (H)C(CO)NH (2048 × 40 × 96) and ^15^N-edited TOCSY-HSQC (2048 × 40 × 128) (Sattler et al. 1999) spectra. 2D ^1^H-^15^N HSQC spectra were collected after each triple-resonance experiment to ensure sample stability over the course of time.

All NMR data were processed on an Intel PC workstation running openSuse Leap 15.1 using NMRPipe/NMRDraw (Delaglio et al. 1995). The ^1^H, ^13^C, and ^15^N chemical shifts were referenced to the internal standard DSS using IUPAC-IUB recommended protocols (http://www.bmrb.wisc.edu/ref_info/cshift.html). Spectra were visualized and analyzed using CCPNMR analysis (Vranken et al. 2005). Assignments were made manually. Secondary structure was deduced from the chemical shift values of ^1^H^N^, ^15^N, ^13^C^α^, ^13^C^β^, and ^13^C’ atoms in the program CSI (chemical shift index) v 3.0 (Hafsa et al. 2015).

The ^1^H^N^ and ^15^N chemical shifts of KRAS4b^T35S/C118S^-GppNHp and KRAS4b^T35S/Q61L/C118S^-GppNHp were compared by using the weighted-average chemical shifts (∆δ_weighted_) from the equation: [∆δ = {(∆H)^2^ + (∆N/5)^2^}^0.5^]. A cut-off value of 0.146 ppm (one standard deviation above the average value) was used in the data analysis. Overlay of 2D ^1^H–^15^N HSQC spectra (700 MHz data) was analyzed in CCPNMR and in NMRDraw. Data were collected and processed in an identical manner for both the protein constructs.

The homology modeling of the KRAS^T35S/C118S^-GppNHp structure was performed with Modeller (v9.24) (Webb and Sali, 2016) using the template structure of the HRAS^T35S^-GppNHp solution structures (RCSB ID 2LCF). The structure model with the lowest DOPE score was chosen as representative structure of K-Ras^T35S/C118S^-GppNHp. Figure 3B was prepared in PyMOL (The PyMOL Molecular Graphics System, Version 2.0 Schrodinger, LLC).

## Extent of assignments and data deposition

**Fig. 1:**
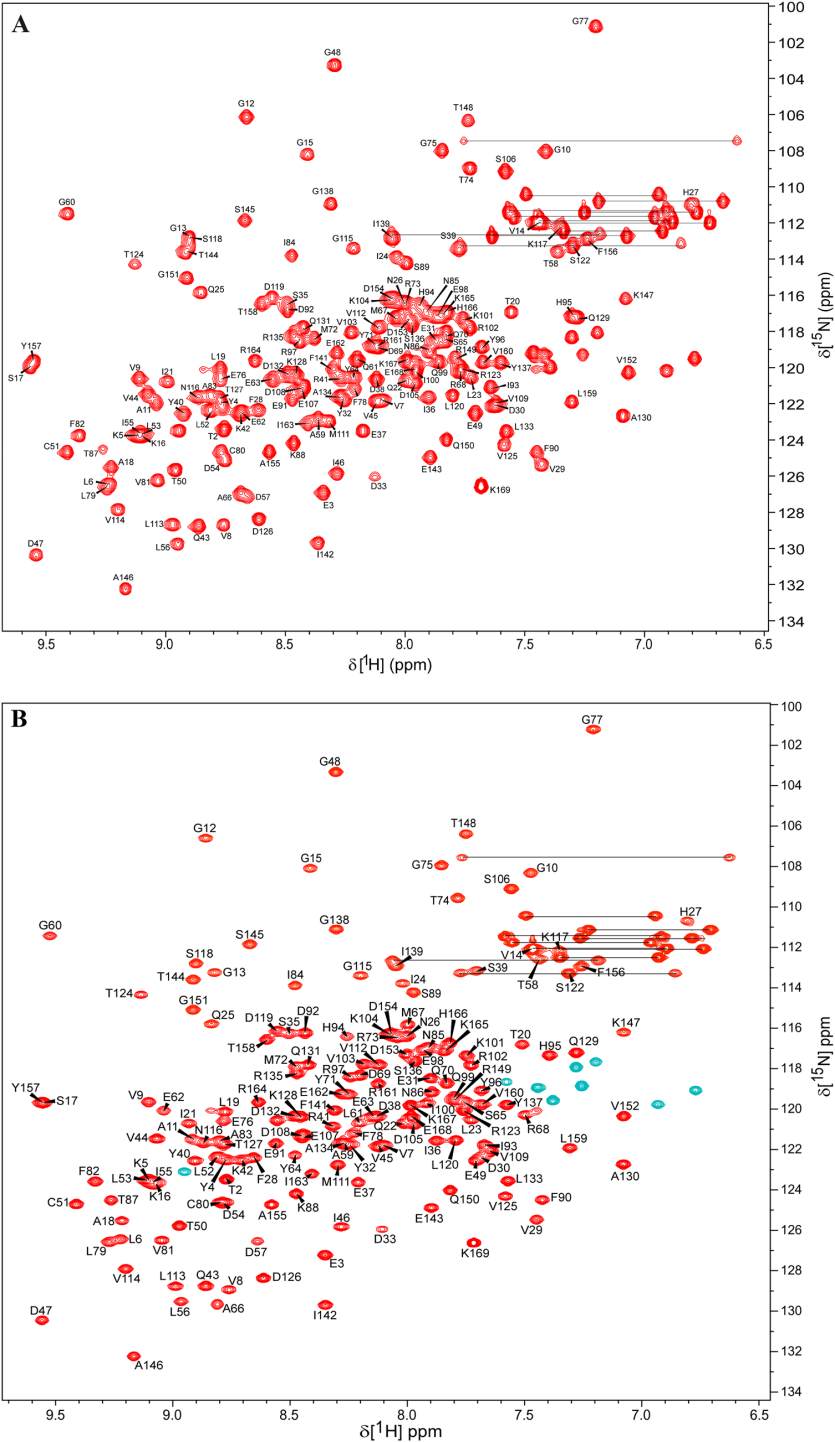
Two-dimensional ^1^H–^15^N HSQC spectrum illustrating the AA residue assignments of **A** KRAS^T35S/C118S^-GppNHp and of **B** KRAS^T35S/Q61L/C118S^-GppNHp at pH 6.5. Spectra were recorded on Bruker Avance spectrometers at 298 K. The assignments shown are annotated using the one letter amino acid code followed by the sequence number of that residue. The unassigned side-chain N–H correlations of Asn, Gln (connected by horizontal lines), and Arg are also seen. Arg side-chain N–H correlations of KRAS^T35S/Q61L/C118S^-GppNHp are shown in green in **B**

The ^1^H, ^13^C, and ^15^N, chemical shifts assignments of KRAS^T35S/C118S^-GppNHp and KRAS^T35S/Q61L/C118S^-GppNHp have been deposited into BMRB (http://www.bmrb.wisc.edu/). Assigned 2D ^1^H–^15^N HSQC spectrum of KRAS^T35S/C118S^-GppNHp and of KRAS^T35S/Q61L/C118S^-GppNHp are shown in Fig. 1. A good dispersion of ^1^H–^15^N correlation cross-peaks as witnessed in the 2D ^1^H–^15^N HSQC spectra of KRAS^T35S/C118S−^GppNHp (Fig. 1A) and of KRAS^T35S/Q61L/C118S−^GppNHp (Fig. 1B) indicates that these proteins adopt a well-folded conformation in solution. High resolution NMR data allowed unambiguous chemical-shift assignments for a significant number of residues. A total of 164 out of 165 non-proline ^1^H–^15^N correlation peaks (99.4%) were identified and assigned for both the proteins, except for their first methionine residue that could not be visualized presumably due to the exchange of its ^1^H^N^ proton with the bulk solvent. For KRAS^T35S/C118S^-GppNHp, ^13^C^α^ peaks for all 169 residues were assigned. ^13^C^β^ signals were not observed for Ser17, Thr20, and Thr158 (155 out of 158 were observed and assigned), whereas ^13^C’ (carbonyl) peaks could not be assigned for the 4 residues preceding prolines (Asp33, Val109, Leu120, and Ile139) and for Lys169 due to unavailability of HN(CA)CO data. KRAS^T35S/Q61L/C118S^-GppNHp also exhibited well resolved spectral data. All 169 resonances of the ^13^C^α^ peaks of the Q61L protein were assigned. As noted in non-Q61L counterpart, ^13^C^β^ signals were not detected for residues Ser17, Thr20, and Thr158 (98.1% completion). ^13^C’ peaks, on the other hand, were assigned for all residues, except for Ile93. Overall, the T35S mutation enables the observation of ^1^H–^15^N correlation cross-peak for residues at positions 7, 10, 12, 13, 16, 21, 22, 29, 30–33, 35–42, 54–55, 57–73, 76, 94 in the 2D ^1^H–^15^N HSQC spectrum, which are usually absent in the data collected at room temperature and near the solution pH used here.

**Fig. 2:**
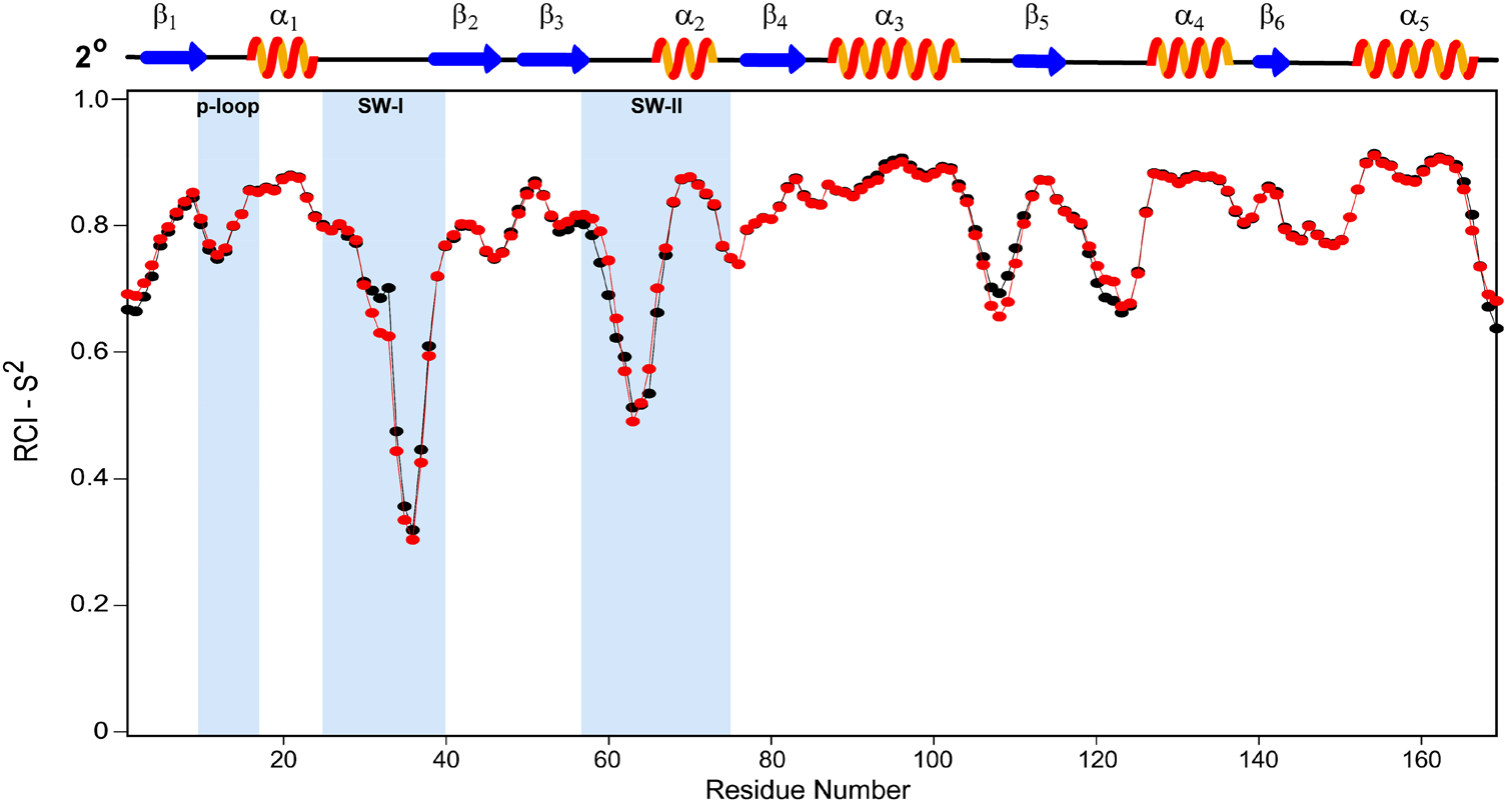
The RCI-S^2^ order-parameter prediction of amino acid residues of KRAS^T35S/C118S^-GppNHp (in black) and of KRAS^T35S/Q61L/C118S^-GppNHp (in red) as deduced from assigned chemical shifts (^13^C^α^, ^13^C^β^, ^13^C^'^, ^15^N, ^1^H^N^) in TALOS-N is shown. Flexible structural regions encompassing p-loop, SW-I, and SW-II are highlighted in light blue background. Shown on top are the secondary structured (2°) elements (five α-helices and six β-strands) present in both the proteins. Secondary structure is determined from the consensus between the results of CSI and of high-confidence values as interpreted by TALOS-N

Regions of secondary structure in both protein conformations were identified from the ^1^H, ^15^N, ^13^C^α^, ^13^C^β^, and ^13^C’ chemical shifts in the programs Chemical Shift Index (CSI) v 3.0 (Hafsa et al. 2015) and TALOS-N (Shen and Bax 2013). Results show that the protein conformations of both K-RAS^T35S/C118S^-GppNHp and KRAS^T35S/Q61L/C118S^-GppNHp are comprised of a mixed distribution of 5 α helices and 6 β strands (Fig. 2). These 11 canonical secondary structure elements are arranged in the order of β_1_-α_1_- β_2_- β_3_- α_2_- β_4_- α_3_- β_5_- α_4_- β_6_- α_5_ that aligns with those noted for HRAS^T35S^-GppNHp (Araki et al. 2011) and is a well-conserved feature of the KRAS4b fold, in general. A consensus between the results of CSI and high-confidence values as interpreted by TALOS-N were used to deduce the residues encompassing these structured elements of the K-Ras^T35S/C118S^-GppNHp as β 1 (3–10), α 1 (16–24), β 2 (39–46), β 3 (50–57), α 2 (66–73), β 4 (77–83), α 3 (87–103), β 5 (111–115), α 4 (127–136), β 6 (140–143), α 5 (152–166) (Fig. 2). A similar secondary structure behavior is shown by the conformation of KRAS^T35S/Q61L/C118S^-GppNHp.

Random Coil Index Order Parameter value (RCI-S^2^), as produced by TALOS-N, represents the conformational rigidity adopted by the particular residue in a protein structure. Such a plot of RCI-S^2^ of KRAS^T35S/C118S^-GppNHp and of KRAS^T35S/Q61L/C118S^-GppNHp residues is shown in Fig. 2. An S^2^ value of ≥ 0.8 adopted by the above-mentioned structured elements is reflective of a relatively rigid backbone conformation of such residues, in contrast to the flexible loop regions (significantly lower S^2^ value). Notably, the loops in the SW-I and SW-II regions are relatively more flexible than those loops in other parts of protein conformation in both the cases. AA residues 31–38 in SW-I region exhibit relatively lower values for KRAS^T35S/Q61L/C118S^-GppNHp than noted for KRAS^T35S/C118S^-GppNHp (Fig. 2), and suggest that these residues adopt a relatively more flexible protein conformation in the former. In contrast, such a comparison for residues 57–68 (except for the residues Glu62 and Glu63) in SW-II elicits a less flexible backbone conformation for these residues in the double mutant (T35S/Q61L) protein as compared to that in the single mutant (T35S). Excluding the terminal residues, other regions of differences between the two proteins include the loop residues 107–111 and 119–123 that show relatively more flexible protein conformations in the Q61L/T35S mutant and in the T35S mutant, respectively.

**Fig. 3:**
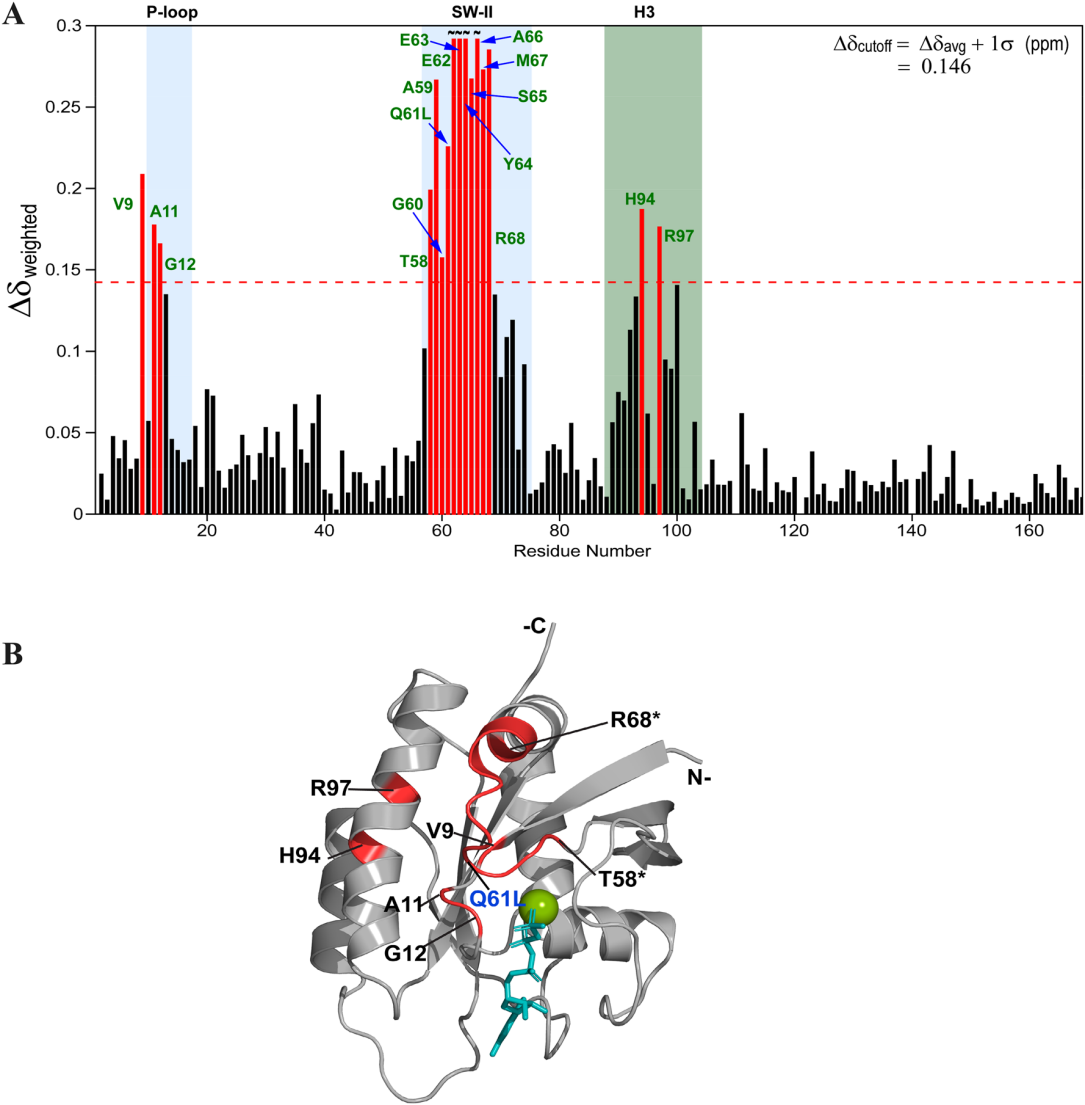
**A** The plot of ^1^H^N^ and ^15^N chemical shift difference (∆δ_weighted_) between KRAS^T35S/C118S^-GppNHp (BMRB ID 50651) and KRAS^T35S/Q61L/C118S^-GppNHp (BMRB ID 50652) *vs* residue number (*see methods for details*). Significant difference in ∆δ of highlighted sixteen residues (rendered red) between the two proteins likely reflect the Q61L mutation induced conformational changes. Threshold value (∆δ_cutoff_) is represented as horizontal dashed line (shown in red). **B** Q61L-mutation induced conformational differences (in red) from WT protein (both in T35S background) are rendered onto the structure model of T35S mutant of KRAS4b (*see methods for details)*. T58* and R68* represent the termini residues eliciting CSP in SW-II region. GppNHp and Mg^2+^ are rendered teal and lime, respectively. Figure is prepared in PyMOL

Next, we examined the effect of the presence of Q61L oncogenic mutation on the protein conformation by comparing the 2D ^1^H–^15^N HSQC spectra of KRAS^T35S/C118S^-GppNHp and KRAS^T35S/Q61L/C118S^-GppNHp. Analysis of chemical shift perturbation (CSP), as carried out from the spectral overlay, identifies sixteen residues exhibiting significant perturbations due to the Q61L mutation. As shown in Fig. 3A, these residues belong to the P-loop (Val9, Ala11, and Gly12), SW-II (Thr58-Arg68), and helix α 3 (His94 and Arg97). Notably, SW-II perturbations were larger among the 3 perturbed regions, and the residues Glu62-Tyr64, and Ala66 showed much larger perturbations in the group. These Q61L induced conformational differences are mapped onto the structural model of KRAS^T35S/C118S^-GppNHp (Fig. 3B). Interestingly, CSP residues at the positions 9, 11, and 12 in the P-loop, and 58–61 and 64–68 in SW-II regions show relatively lower RCI-S^2^ values in KRAS^T35S/C118S^-GppNHp to that in KRAS^T35S/Q61L/C118S^-GppNHp, suggesting that the local protein conformation adopted by these residues is relatively less flexible in the oncogenic mutant. Residues Glu62 and Glu63, on the other hand, elicit a more flexible backbone conformation in the oncogenic mutant (Fig. 2).

Although it is apparent that Leu61 is structurally proximal to residues Gly60, Glu62, Glu63, and Ala11, the chemical shift differences for other P-loop and switch-2 residues (Tyr64-Arg68; Thr58, Ala59), and α 3 residues are noteworthy. It would be interesting to understand how the local conformations of the Leu61 and other perturbing residues are packed with respect to each other in the three-dimensional solution structure of KRAS^T35S/Q61L/C118S^-GppNHp, and whether the SW-II region conformation (encompassing the perturbed residues) is oriented away from, or packs closer to helix α 3 in solution. These structural investigations will also inform about the conformational variability of the SW-II region attributed to the Q61L mutation, and thus be helpful in characterizing the size(s) of the potential pockets involving the SW-II region (SOS-, effector-, SW-II/ α 3) in this oncogenic construct vs that present in the non-Q61L counterpart protein. Moreover, as the residues Gly60-Tyr64 are mostly disordered in the crystal structures of the active form of oncogenic K-RAS (including the Q61L mutant), the solution structure of KRAS^T35S/Q61L/C118S^-GppNHp would provide clues to the conformational packing elicited by these residues.

In conclusion, the backbone assignments of KRAS^T35S/C118S^-GppNHp and KRAS^T35S/Q61L/C118S^-GppNHp reported here will be helpful to future NMR studies pertaining to drug discovery efforts that involve identification of ligand binding sites for small molecules with detectable affinity regime. Q61L KRAS-GTP exhibits significantly lower rates of intrinsic hydrolysis among the common oncogenic KRAS mutants (Hunter et al. 2015). The Leu61 induced CSP determinants identified here in conjunction with three-dimensional structures could be further exploited in such functional studies of the active KRAS.

## Supplementary Information

Below is the link to the electronic supplementary material.

Supplementary file1 (DOCX 11 kb)

## Data Availability

The assignments have been deposited to the BMRB with accession codes 50,651 (T35S) and 50,652 (T35S/Q61L).
