# Supplementary material for: NMR 1H, 13C, 15N backbone resonance assignments of the T35S and oncogenic T35S/Q61L mutants of human KRAS4b in the active, GppNHp-bound conformation
Source: Biomol NMR Assign. 2021 Oct 22;16(1):1–8. doi: 10.1007/s12104-021-10050-7 (PMC9068649; doi:10.1007/s12104-021-10050-7)
Supplement: Supplementary file 1 — Supplementary file1 (DOCX 11 kb) [file 12104_2021_10050_MOESM1_ESM.docx]

<http://deposit.bmrb.wisc.edu/author_view/BMRB/50651_hy_plxwhezb.str>

<http://deposit.bmrb.wisc.edu/author_view/BMRB/50652_hy_sjbbmjre.str>
